# Chikungunya virus molecular evolution in India since its re-emergence in 2005

**DOI:** 10.1093/ve/veab074

**Published:** 2021-08-25

**Authors:** Sakshi Chaudhary, Jaspreet Jain, Ramesh Kumar, Jatin Shrinet, Scott C Weaver, Albert J Auguste, Sujatha Sunil

**Affiliations:** Vector Borne Diseases Group, International Centre for Genetic Engineering and Biotechnology, Aruna Asaf Ali Marg, New Delhi 110067, India; Vector Borne Diseases Group, International Centre for Genetic Engineering and Biotechnology, Aruna Asaf Ali Marg, New Delhi 110067, India; World Reference Center for Emerging Viruses and Arboviruses, University of Texas Medical Branch, Galveston, TX 77555, USA; Department of Microbiology and Immunology, University of Texas Medical Branch, Galveston, TX 77555, USA; Institute for Human Infections and Immunity, University of Texas Medical Branch, Galveston, TX 77555, USA; Vector Borne Diseases Group, International Centre for Genetic Engineering and Biotechnology, Aruna Asaf Ali Marg, New Delhi 110067, India

**Keywords:** chikungunya virus (CHIKV), whole-genome sequencing, phylogeny, variants

## Abstract

Chikungunya virus (CHIKV), an alphavirus of the *Togaviridae* family, is among the most medically significant mosquito-borne viruses, capable of causing major epidemics of febrile disease and severe, chronic arthritis. Identifying viral mutations is crucial for understanding virus evolution and evaluating those genetic determinants that directly impact pathogenesis and transmissibility. The present study was undertaken to expand on past CHIKV evolutionary studies through robust genome-scale phylogenetic analysis to better understand CHIKV genetic diversity and evolutionary dynamics since its reintroduction into India in 2005. We sequenced the complete genomes of fifty clinical isolates collected between 2010 and 2016 from two geographic locations, Delhi and Mumbai. We then analysed them along with 753 genomes available on the Virus Pathogen Database and Analysis Resource sampled over fifteen years (2005–20) from a range of locations across the globe and identified novel genetic variants present in samples from this study. Our analyses show evidence of frequent reintroduction of the virus into India and that the most recent CHIKV outbreak shares a common ancestor as recently as 2006.

## 1. Introduction

The *Alphavirus* genus is a diverse group of small, spherical, enveloped viruses with single-stranded, positive-sense RNA genomes (Forrester et al. 2012; Lanciotti and Lambert 2016; Villero-Wolf et al. 2019). These viruses infect vertebrates such as primates, fish, and birds and are medically important owing to their ability to cause diseases in humans. Chikungunya virus (CHIKV) is among the most important of these human-pathogenic alphaviruses. It is known to cause epidemics of chikungunya fever (CHIKF), an acute febrile illness characterized by sudden onset of fever, rashes, headache, nausea, and muscle pain, and usually associated with severe, debilitating joint pain and swelling (Ganesan, Duan, and Reid 2017). The virus is transmitted by *Aedes* mosquitoes, including in urban settings, and is a significant public health concern in India. Isolated first during the 1952–3 epidemics of dengue-like illness from a patient in Tanganyika (now Tanzania), CHIKV epidemics were subsequently reported in many parts of Africa. The first report of CHIKV in Asia surfaced during the late 1950s with an incidence rate of 46 per cent (India), 9 per cent (Thailand), and 6.81 per cent (Malaysia) (Wimalasiri-Yapa et al. 2019).

The CHIKV genome is about 11 kb in size and encodes the structural (C, E3, E2, 6K/TF, and E1) and non-structural (nsP1-4) proteins through two separate open reading frames. Structural and non-structural proteins are translated as separate polyproteins and, subsequently, catalytically cleaved to the individual proteins. Furthermore, the genome is flanked by 5′ and 3′ untranslated regions (Hyde et al. 2015). Recent reports have identified mutations in the structural and non-structural proteins of CHIKV worldwide that can lead to changes in disease dynamics (Sasmono et al. 2017; Wong and Chu 2018). While mutations within the structural proteins affect vector specificity, epidemic potential, host response, and disease progression, variations in the non-structural proteins are responsible for altered CHIKV replication kinetics (Fros et al. 2010; Munoz-Medina et al. 2018).

Phylogenetic studies of CHIKV have demonstrated that the virus exists as three major, originally geographically distinct genotypes: Asian, East/Central/South African (ECSA), and West African (WA). ECSA CHIKV strains have caused most outbreaks and epidemics in recent times, and the availability of a large number of sequences, in addition to their in-depth analysis, has enabled their further segregation into distinct lineages, such as the Indian Ocean lineage (IOL) (Chen et al. 2016; Xavier et al. 2019). Times to most recent common ancestors (tMRCA) estimated using Bayesian coalescent analysis determined that Asian and ECSA lineages diverged around 100 years ago, but their earlier divergence from the WA was unclear until recently (Volk et al. 2010; Nasci 2014). Although CHIKV ECSA strains are considered highly virulent due to their scale of spread and Severity of chronic disease during recent epidemics, studies on viral pathogenesis in murine models revealed WA strains to be the most pathogenic followed by ECSA and Asian strains (Langsjoen et al. 2018; Jain et al. 2020).

In India, the first major CHIKF outbreak was reported in 1963 in Kolkata, West Bengal (Shah, Gibbs, and Banerjee 1964), and the disease spread to other states of India between 1964 and 1973 (Pavri 1986; Yadav et al. 2003). During this period, the strain that prevailed belonged to the Asian genotype (Yergolkar et al. 2006). After 1973, CHIKF disappeared from the Indian subcontinent until its re-emergence in 2005, when large-scale outbreaks were reported in several parts of India, confirming widespread re-emergence in the country. Since then, CHIKV transmission has continued sporadically in various parts of the country. Since the 2005 CHIKF emergence, the causative strain for outbreaks in India has been the ECSA strain, IOL (Arankalle et al. 2007; Chhabra et al. 2008; Dwibedi et al. 2011; Dikid et al. 2013).

CHIKV in India has unique epidemiological dynamics due to various factors: the presence of both urban vectors, *Aedes*  *aegypti* and *Aedes*  *albopictus*, distinct geographical distribution patterns of these vectors in the country, varying rainfall and temperature patterns, high migration rates of human populations within the country, and a steady inflow of travellers from other endemic geographical locations across the globe. These factors make studying CHIKV evolution within the country tedious and challenging. The present study was undertaken to infer the spatiotemporal evolution of CHIKV in India and to better define its evolutionary dynamics and genetic diversity. We studied CHIKV clinical strains collected from two geographically distinct locations with distinct disease patterns over a period of six years. The two geographical locations Mumbai and Delhi have similar total populations but distinct geographical conditions (Jain et al. 2017), as well as epidemiologic characteristics; Mumbai reported CHIKF since the 1970s with different circulating virus strains (Arankalle et al. 2007), whereas Delhi reported its first case of CHIKF in 2006, with the ESCA CHIKV strain as the causative agent (Chahar et al. 2009). Whole genomes of viruses were sequenced and genome-scale phylogenetic and evolutionary analyses of mosquito- and human-derived CHIKV strains from throughout India between 2005 and 2020 were conducted using whole genomes deposited in the Virus Pathogen Database and Analysis Resource (ViPR).

## 2. Results

### 2.1 Whole-genome sequencing and assembly

A total of fifty confirmed CHIKV-positive sera were processed for whole-genome sequencing and further analysis (Table 1). Upon assembly of the complete set of samples, a total of 758.7X coverage was obtained. The coverage ranged from 0.08X (IND/2016/DEL/08) to 8,201.95X (IND/2011/MUM/02). Those regions with gaps were filled using RT-PCR and Sanger amplicon sequencing to complete the genome assembly.

**Table 1.** Summary of clinical samples used for whole-genome sequencing and analysis.

S. No.	Sample nomenclature^a^	Collection year	Number of reads	Assembly coverage	Accession number
1.	IND/2010/MUM/02	2010	24,157,872	4,104.28X	MW581870
2.	IND/2010/MUM/40	2010	26,684,617	992.48X	MW581884
3.	IND/2010/DEL/01	2010	39,784,164	890.20X	MH124570
4.	IND/2010/DEL/03	2010	37,069,923	52.09X	MH124571
5.	IND/2010/DEL/04	2010	35,792,022	84.19X	Omitted from analysis
6.	IND/2010/DEL/05	2010	39,497,274	78.40X	MH124572
7.	IND/2010/DEL/06	2010	46,996,466	28.46X	MH124573
8.	IND/2010/DEL/07	2010	33,558,891	4.29X	MW581865
9.	IND/2010/DEL/10	2010	29,227,869	8.66X	MH124574
10	IND/2010/DEL/11	2010	37,350,131	372.40X	MH124578
11.	IND/2010/DEL/12	2010	32,601,265	40.92X	MH124579
12.	IND/2010/DEL/13	2010	28,341,978	24.13X	MH124575
13.	IND/2010/DEL/14	2010	25,845,213	15.13X	MH124576
14.	IND/2010/DEL/20	2010	22,916,619	10.16X	MH124577
15.	IND/2010/DEL/48	2010	80,522,715	5.73X	MW581866
16.	IND/2010/DEL/88	2010	40,664,171	10.98X	MW581863
17.	IND/2010/DEL/108	2010	41,490,431	2,746.96X	MW581868
18.	IND/2011/MUM/02	2011	28,163,124	8,201.95X	MW581871
19.	IND/2011/MUM/320	2011	26,636,982	6,571.79X	MW581885
20.	IND/2011/DEL/01	2011	34,284,491	29.23X	MW581864
21.	IND/2011/DEL/11	2011	89,587,490	5,720.75X	MW581872
22.	IND/2011/DEL/12	2011	77,059,273	41.74X	Omitted from analysis
23.	IND/2012/DEL/01	2012	28,364,398	5.30X	Under process
24.	IND/2012/DEL/02	2012	33,493,890	0.13X	Omitted from analysis
25.	IND/2012/DEL/08	2012	27,465,973	14.41X	MW581873
26.	IND/2012/DEL/09	2012	30,563,321	52.49X	MW581874
27.	IND/2012/DEL/15	2012	32,245,250	20.19X	MW581881
28.	IND/2012/MUM/32	2012	23,947,739	6,869.40X	MW581883
29.	IND/2012/MUM/33	2012	24,457,761	3,690.93X	Under process
30.	IND/2012/MUM/39	2012	17,792,875	7,709.12X	MW581876
31.	IND/2012/MUM/48	2012	31,808,240	1,667.08X	MW581867
32.	IND/2012/MUM/53	2012	30,679,791	4978.09X	MW581875
33.	IND/2012/MUM/54	2012	22,469,392	16.74X	Under process
34.	IND/2013/DEL/59	2013	67,863,917	135.60X	MW581862
35.	IND/2013/MUM/103	2013	27,068,500	5,762.31X	MW581880
36.	IND/2013/MUM/133	2013	30,149,059	5,377.81X	MW581882
37.	IND/2013/MUM/135	2013	36,773,329	4,729.80X	MW581869
38.	IND/2013/MUM/136	2013	25,455,983	5,524.58X	MW581878
39.	IND/2013/MUM/174	2013	25,069,560	4,599.55X	MW581877
40.	IND/2016/DEL/01	2016	41,946,797	44.89X	MH124580
41.	IND/2016/DEL/02	2016	35,696,953	17.48X	MH124581
42.	IND/2016/DEL/03	2016	40,181,704	48.10X	MH124582
43.	IND/2016/DEL/04	2016	34,345,555	0.34X	Omitted from analysis
44.	IND/2016/DEL/05	2016	34,470,436	1.26X	Omitted from analysis
45.	IND/2016/DEL/06	2016	42,592,594	0.70X	Omitted from analysis
46.	IND/2016/DEL/07	2016	40,524,008	1.77X	Omitted from analysis
47.	IND/2016/DEL/08	2016	33,072,469	0.08X	Omitted from analysis
48.	IND/2016/DEL/09	2016	40,603,013	3.19X	Omitted from analysis
49.	IND/2016/DEL/10	2016	35,368,129	1.97X	Omitted from analysis
50.	IND/2016/DEL/11	2016	28,328,222	8.31X	MH124583

a Nomenclature of sample ID: IND refers to the country of the sample, i.e. India. The four-digit number after slash refers to the year of collection. DEL and MUM refer to the regions of collection, i.e. MUM is Mumbai and DEL is Delhi. The two-digit number after slash refers to the sample number in the order of collection for that specific year and location.

These fifty samples were evaluated on the basis of coverage, and those sequences showing less than 4× coverage were removed from further analyses. Furthermore, the sequences were analysed for recombination events and five sequences, namely IND/2010/DEL/04, IND/2011/DEL/12, IND/2016/DEL/02, IND/2016/DEL/09, and IND/2016/DEL/10, were potential recombinant and thus removed from further analysis (data not shown). On the basis of the above criteria, a final set of forty-one CHIKV whole-genome sequences was taken for downstream analyses.

### 2.2 Phylogeography of CHIKV populations

**Figure 1.**
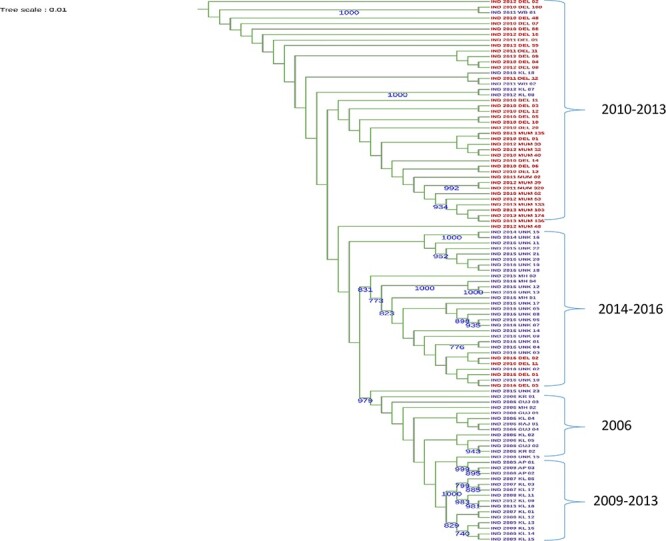
The ML phylogenetic (midpoint rooted) tree of Indian CHIKV strains using complete genome sequences (*n* = 99). Numbers adjacent to branches show bootstrap values >70 per cent. Red colour nodes represent sequences from the present study. Sequences downloaded from the public database are coloured in violet.

Phylogenetic analysis of all complete CHIKV genome sequences was performed using two sequence data sets obtained from the ViPR on the basis of their geographical locations and year of sample collection. One data set consisted of ninety-nine sequences from India only, including fifty-eight sequences from public databases, and the other data set was generated using 794 sequences from strains present across the globe belonging to all the three CHIKV genotypes, including 753 sequences from the public database (Fig. 1 and Supplementary Fig. S2). Phylogenetic analysis of the forty-one samples using the global strains revealed that all 2010–6 Indian samples belonged to the ECSA genotype. Detailed analysis of these samples that were collected over a span of six years with the global sequences revealed a distinct clustering (Supplementary Fig. S2). The samples from 2010 to 2013 clustered together with all the 2010 samples, and the 2012 and 2013 samples formed a subcluster within this clade. The Indian samples from 2016 clustered separately along with samples from Hong Kong, Singapore, Kenya, and Pakistan collected in 2015–6 (Supplementary Fig. S2). These findings suggest a possible reintroduction of CHIKV into India around 2015–6, leading to the major outbreak in 2016.

Additionally, we determined the phylogenetic relationships among CHIK viruses within India since 2006 (*n* = 99). Phylogenetic trees based on complete genome sequences were inferred using the maximum likelihood (ML) and the Markov chain Monte Carlo methods with bootstrap values of >70 (Fig. 1). The strains clustered into distinct temporal groups that were in some instances geographically distinct. Viruses sampled during 2010–3 clustered together, whereas the 2014–6 samples clustered into a different group. Samples of 2006 were from different states; however, they clustered together suggesting a common source, probably that of the initial 2006 re-emergence. Geographically close regions shared the same CHIKV sublineage in the later years, as was seen in Kerala and Andhra Pradesh. Most of the samples of these regions during 2009–13 clustered together, suggesting that CHIKV lineage variation is a major underlying determinant of epidemics. Tests of neutrality using Tajima’s *D* test revealed an excess of low-frequency variants that might have resulted from population expansions.

Estimates of average evolutionary divergence over sequence pairs within Indian states showed varying divergence both within and between states. Sequences derived from Kerala and Maharashtra showed maximum divergence, suggesting the existence of an ancestral population in Maharashtra until 2009. From 2010, similar evolutionary differences were observed in both Delhi and Maharashtra samples until 2013, and sequences from Delhi collected in the year 2016 were a part of a separate clade and thus were most divergent from the rest of the Indian samples. These findings suggest the emergence of distinct virus clades in the two geographical regions.

### 2.3 Evolutionary history of Indian CHIKV genetic groups

**Figure 2.**
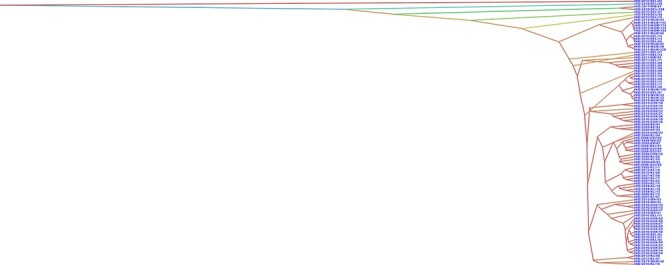
The DensiTree showing the uncertainty between isolates. In this tree set, there are seven clearly distinguishable clades with different colours, showing large uncertainty of the topologies. Further details such as node ages and tree scale are provided in Supplementary Fig. S3.

To infer the time scale of the emergence of CHIKV genotypes, we estimated the tMRCA. The evolutionary rates of each defined genetic group were derived based on maximum clade credibility (MCC) phylogeny under an uncorrelated log-normal relaxed molecular clock. The tree highlights the uncertainty within clades but the split at the root into two groups clearly shows the diversity within the samples. Collectively, the phylogenetic structure and tMRCA estimates of the CHIKV genotypes suggested that there has been increased genetic diversity owing to the emergence of fit variants that possibly emerged during the intermittent outbreaks in the country (Fig. 2 and Supplementary Fig. S3). To investigate changes in demographic patterns of CHIKV over time, we estimated the changes in relative genetic diversity using molecular clocks and coalescent models that allow the estimation of evolutionary rates and timescales. The MCC tree revealed a clustering pattern similar to that of the phylogenetic analysis as aforementioned and further indicated that the samples from the recent outbreaks shared the most common ancestor from the year 2006 (95 per cent Bayesian credible interval = 2005–20; Supplementary Table S1).

### 2.4 Amino acid variability analysis

Previous studies have shown that single amino acid substitutions at critical sites of CHIKV proteins have influenced the epidemiology of the virus as observed with E1-A226V, which enabled enhanced transmission by *Ae.*  *albopictus*, a more globally dispersed vector, especially in temperate regions (Tsetsarkin et al. 2007). Another substitution, E2-L210Q, identified in the CHIKV populations of Kerala State, India, conferred a selective advantage by increasing the initial infection of *Ae. albopictus* midgut epithelial cells and subsequent CHIKV dissemination into the haemocoel, thereby promoting transmission by this vector (Tsetsarkin and Weaver 2011). Our complete coding sequence alignment revealed conservative and non-conservative amino acid substitutions in each major CHIKV protein. Even among those conservative amino acid substitutions that were not statistically significant in the entropy analysis, there were rapidly evolving variations (Supplementary Table S2). Overall, entropy analysis identified a total of thirteen codon positions with a high score (*P* < 0.05), indicating a higher possibility of tolerated variations at these positions.

### 2.5 Variant analysis

**Figure 3.**
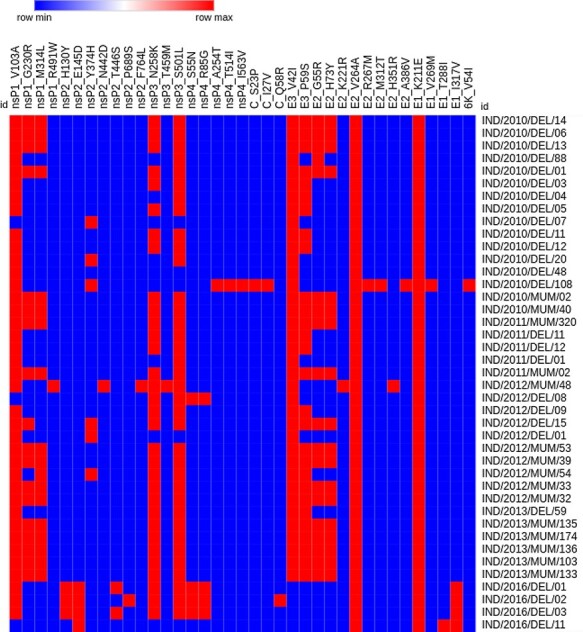
Hierarchical clustering of non-synonymous mutations in CHIK samples. A total of thirty-nine non-synonymous mutations was observed in the structural (E1, E2, E3, C, and 6K/TF) and non-structural (nsP1, nsP2, nsP3, and nsP4) protein genes in a total of forty-one samples collected between 2010 and 2016. The blue colour represents the absence of mutation in the respective sample, while the red colour shows the mutation’s presence.

Mutation analysis of our forty-one CHIKV strains against a reference strain collected in 2006 from India (FJ000068) revealed a total of 211 mutations. Of these, thirty-nine mutations were non-synonymous, including twenty amino acid variants in the non-structural protein genes and nineteen in the structural genes (Fig. 3), with nsP1 the most variable. Sample-wise analysis of the mutations revealed that the IND/2010/DEL/108 strain carried a maximum of thirteen mutations, of which ten (Supplementary Table S3) were unique. Likewise, strain IND/2016/DEL/02 was found to contain twelve mutations, of which nsP2-P689S and C-Q58R were unique (Fig. 3). In addition to these substitutions, we detected a frameshift in sample IND/2012/MUM/54 at position 98 caused by the deletion of A and T nucleotides, corresponding to frameshift nsP1-I8 at the amino acid level. Mutations identified from this study are represented in Supplementary Table S3.

#### 2.5.1 Annual analysis of sequences

**Figure 4.**
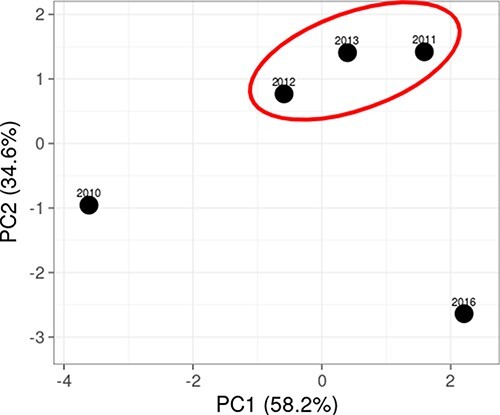
Principal component analysis of 2010, 2011, 2012, 2013, and 2016 CHIKV strains.

We attempted to understand the status of the mutations in our samples over the years since the 2005 CHIKV re-emergence in India. The samples from the present study collected from five different years (sixteen sera were collected in 2010, five in 2011, eleven in 2012, six in 2013, and four in 2016) were further analysed, revealing that the frequency of non-synonymous mutations was higher in structural protein genes as compared to non-structural in most of the CHIKV strains. The analysis further revealed that in non-structural proteins, four non-synonymous mutations (Supplementary Table S3) were common to all samples in certain years. Substitutions nsP1-G230R, nsP1-M314L, nsP2-Y374H, E3-V42I, E3-P59S, E2-G55R, and E2-H73Y were present in samples from 2010 to 2013; however, these mutations were absent in the 2016 samples. Likewise, seven mutations were present only in 2016 samples and not in any of the previous years. Mutation nsP4-S55N was seen in one sample collected from Delhi in 2012. We did a principal component analysis to understand the year-wise relationship between CHIKV strains (Fig. 4; Leigh, Bryant, and Nakagawa 2015). Strains from 2010 and 2016 formed separate groups coinciding with major outbreaks in these respective years, while strains from 2011, 2012, and 2013 grouped together coinciding with episodic occurrences.

#### 2.5.2 Regional analysis of sequences

Our analysis further revealed distinct mutations that may have been positively selected within the two main regions of our sample collections, i.e. Delhi and Mumbai. We analysed these mutations based on their presence in samples from these geographical locations. For the sake of unbiased comparison, samples collected from Delhi in 2016 were not included in the analysis as we did not have samples from Mumbai during that year. Our analysis revealed that thirty-one non-synonymous mutations were present in the isolates collected from Delhi, with sixteen present in structural protein genes and fifteen in non-structural genes. In addition, nineteen non-synonymous mutations were identified in samples collected from the Mumbai region, of which eight were present in structural genes and ten in non-structural proteins.

**Figure 5.**
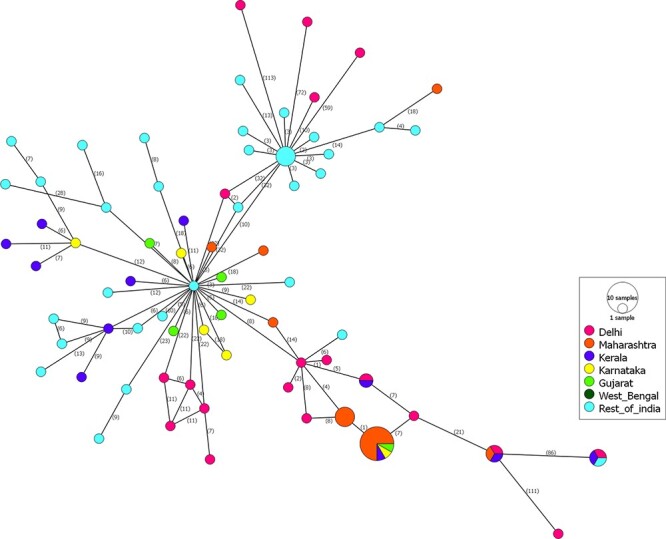
Median joining network analysis to represent region-wise mutations in CHIKV strains. The numbers between strains show the frequencies of mutations. Circles in pink colour represent strains from Delhi, mustard from Maharashtra, blue from Kerala, yellow from Karnataka, light green from Gujarat, forest green from West Bengal, and sky blue from remaining parts of India.

Out of all fifty mutations, nineteen were either present in Delhi or in Mumbai strains. Twelve mutations were unique in Delhi samples, and six were unique in samples from Mumbai (Supplementary Table S3). Mutations nsP1-V103A, nsP1-G230R, nsP1-M314L, nsP2-Y374H, E1-K211E, E2-G55R, E2-H73Y, E2-V264A, E3-V42I, and E3-P59S were present in the isolates collected from both regions. We further observed that during the 2010–3 CHIKV outbreaks, six non-synonymous mutations were only seen in the Mumbai isolates, namely nsP1-R491W, nsP2-N442D, nsP2-F764L, nsP3-T459M, E2-K221R, and E2-H351R. Five non-synonymous mutations were of importance as they were mainly seen only in the Mumbai isolates along with 50 per cent occurrence in the Delhi isolates, namely nsP1-V103A, nsP1-M314L, nsP1-G230R, E1-K211E, and E2-V264A. The results revealed that in Mumbai strains, the mutations were more or less stabilized, whereas Delhi isolates were distributed as two separate groups, with these mutations present in one and absent in the other group. This result provides evidence that the major outbreaks observed in these regions might be attributed to different strains of CHIKV. We constructed a median-joining network of the Indian isolates used in this study using the POPART tool (Leigh, Bryant, and Nakagawa 2015), and it is depicted in Fig. 5. This network was used to visualize genealogical relationships at the intraspecific level. Numbers between strains represent the variation between them.

#### 2.5.3 Epidemic vs. episodic strain mutation analysis

There were two epidemic phases of CHIKV in India in 2010 and 2016. With respect to outbreak-specific mutations, we observed unique mutations in isolates from both outbreaks. Between these years, CHIK occurred as episodic events during 2011–3. To understand the impact of mutations during the outbreaks and episodic events, we compared the inter-episodic mutations with those during the outbreaks of 2010 and 2016. Our analysis revealed that the inter-episodic events clustered together away from the two epidemic outbreaks in 2010 and 2016 (Fig. 4). A total of twenty-seven non-synonymous mutations were present, twelve in non-structural protein genes and fifteen in structural genes (Supplementary Table S3). Interestingly, in all the other samples from 2011 to 2013, six unique mutations, namely nsP1-R491W, nsP2-Y374H, nsP2-N442D, nsP3-T459M, E2-K221R, and E2-H351R, were observed, and these were present only from 2012 samples.

### 2.6 Selection pressure analysis

After identifying mutations, we performed selection pressure analysis of Indian strains to understand the potential impact of mutations on recent outbreaks. Selection pressure analysis performed using all Indian strains (*n* = 99) revealed several sites in the CHIKV genome under purifying selection (Table 2). The low non-synonymous/synonymous (dN/dS) ratio suggested purifying selection in the non-structural gene open reading frame. In total, sixteen amino acids positions were found to be positively selected by the single-likelihood ancestor counting (SLAC) method, fourteen by mixed-effect model of evolution (MEME) method, fourteen by fixed-effect likelihood method (FEL) and twelve by fast, unconstrained Bayesian approximation for inferring selection (FUBAR) method (Table 2).

**Table 2.** Summary of positively selected amino acid positions from the present study.

	SLAC			MEME			FEL			FUBAR		
AA positions	dS	dN	Positively selected (*P* value)	dS	dN	Positively selected (*P* value)	dS	dN	Positively selected (*P* value)	dS	dN	Positively selected (*P* value)
nsP1_103	0.51	6	0.03	0.51	5.24	0.03	0.32	4	0.01	0.46	4.78	0.02
nsP1_160	0	3.89	0.02	–	–	–	–	–	–	–	–	–
nsP1_230	0	5.26	0.05	0	5.24	0.05	0.42	3.21	0.04	0.32	5.43	0.04
nsP2_130	0	2.81	0.08	0	2.81	0.08	0.36	3.16	0.05	0	3.81	0.07
nsP2_442	0	3.69	0.04	0	3.71	0.04	–	–	–	–	–	–
nsP3_258	0	2.64	0.1	0	2.61	0.09	0.62	4.16	0.07	0.21	3.44	0.04
nsP3_501	0	3	0.04	0	3	0.04	0.31	4.04	0.03	0.16	4.21	0.08
nsP4_85	0	2.64	0.06	0	2.64	0.06	0	3.12	0.05	0.22	3.18	0.09
nsP4_102	0	2.86	0.1	–	–	–	0.46	4.10	0.08	0.11	4.91	0.09
nsP4_161	0.81	4.27	0.07	0.81	4.26	0.07	0.31	3.82	0.02	–	–	–
nsP4_250	0	3.28	0.1	0	3.14	0.1	0	4.48	0.06	0.34	4.13	0.1
E3_42	0	2.69	0.09	0	2.69	0.09	0.63	3.84	0.05	0.32	2.14	0.06
E2_73	0	3.34	0.09	0	3.44	0.1	0	5.42	0.09	–	–	–
E2_264	0	3.63	0.1	0	3.54	0.09	0.26	4.48	0.07	0.14	3.79	0.09
E2_351	0	2.29	0.09	0	2.29	0.09	0.17	3.96	0.06	0.21	1.49	0.06
E1_211	0	2.47	0.09	0	2.53	0.09	0	4.57	0.05	0	3.87	0.07

SLAC, single-likelihood ancestor counting; MEME, mixed effects model of evolution; FEL, fixed effect likelihood; FUBAR, fast, unconstrained Bayesian approximation for inferring selection.

## 3. Discussion

We analysed the genetic diversity in the CHIKV genomes obtained directly from the serum samples of patients between 2010 and 2013, and then again in 2016. These years are important, as the pattern of CHIKV outbreaks was quite diverse. The year 2010 witnessed a huge outbreak, and in the following years, there were very few cases (Jain et al. 2017). Thereafter, the year 2016 again saw a huge outbreak (Kaur et al. 2017; Jain et al. 2020). Multiple waves of CHIKV infection have occurred over the past two decades both in India and across the globe, with outbreaks traced from Africa to Southeast Asia, South Asia, and the Americas. Both the Asian and ECSA genotypes have been implicated in these outbreaks (Rezza 2014; Sahadeo et al. 2017). Historically, in India, the Asian genotype was present during the earlier outbreaks in the 1960s and 70s, but the ECSA has been the causative genotype for recent outbreaks, as seen in other Asian countries, such as Malaysia, China, and Indonesia (Dash et al. 2007; Sam et al. 2015; Zeller, Van Bortel, and Sudre 2016). Our phylogenetic analyses reveal that there has been a reintroduction of the virus into the country in addition to local transmission.

Deep sequencing analysis revealed novel insights into the seemingly non-random nature of variants that are present at higher frequencies (>20 per cent) among the geographic population in the country. The fact that a subset of these minor variants is observed across multiple strains is of interest. How these high- and low-frequency variants contribute to viral evolution, host specificity, and/or the host innate and adaptive immune response over time is largely unknown and warrants additional investigation. Previous genomic analyses of CHIKV have reported amino acid substitutions in structural and non-structural genes ranging from two to thirty-two (Stapleford et al. 2016; Silva and Dermody 2017). In the present study, variations in the structural and non-structural genes ranged up to 39, with more mutations observed in the non-structural genes. In the case of the envelope proteins, substitutions such as E1-K211E, E2-G55R, E2-H73Y, E2-V264A, E3-V42I, and E3-P59S have implications for neurovirulence during pathogenesis (Barr and Vaidhyanathan 2019; Cardoso et al. 2019). With respect to non-structural proteins, we observed variants such as nsP1-G230R and nsP3-STOP524R in our samples that have been implicated in increased replication and fitness (Jones et al. 2017; Mounce et al. 2017).

Of special interest are the genetic characteristics among CHIKV isolates in the 2016 outbreak in comparison with those of the outbreaks in previous years. Comparison between the 2016 and 2010 outbreaks have been well characterized in a previous study (Jain et al. 2020); however, the 2016 outbreak shows distinct differences even with the isolates from the years 2011, 2012, and 2013. Our further analysis suggests that the 2016 outbreak could have resulted from a new introduction event rather than from ongoing local transmission. Selection analysis of the viruses from these outbreaks further revealed a low ratio of non-synonymous to synonymous substitution in most comparisons, which may indicate that CHIKV populations in nature are generally subject to strong purifying selection (Zanotto et al. 1996; Yang et al. 2000). Significantly lower rates of nucleotide substitution have been observed in vector-borne compared to other RNA viruses (Jenkins et al. 2002).

In summary, we report a large number of CHIKV complete genome sequences collected from the Delhi and Mumbai regions of India, revealing the epidemiological and evolutionary dynamics of the CHIKV ECSA genotype in India since its first re-emergence in 2005.

## 4. Materials and methods

### 4.1 Samples pre-processing

Recruited patients were part of previous studies funded by the Department of Biotechnology and Department of Science and Technology, Government of India (Jain et al. 2017, 2020; Kaur et al. 2017). Details of samples used in the present study are provided in Table 1. Viral RNA was isolated directly from patient sera using the High Pure Viral Nucleic Acid kit (Product No: 11858874001; Roche, Germany) according to the manufacturer’s instructions. The integrity of RNA was estimated using the bioanalyser, and those samples with an RNA integrity number value of 8 was taken for further processing.

### 4.2 Illumina sequencing

Viral nucleic acid fragmentation, first- and second-strand complementary DNA synthesis and amplification, cluster formation, and paired-end fifty base sequencing on an IlluminaHiSeq 1000 were performed as described previously (Sahadeo et al. 2017). Briefly, viral RNA was fragmented by incubation at 94°C for eight minutes in 19.5 μl of fragmentation buffer (Illumina, Inc., San Diego, CA). First- and second-strand complementary DNA synthesis, adapter ligation, and amplification of the library were performed using the IlluminaTruSeq RNA Sample Preparation kit v2 under conditions prescribed by the manufacturer (Illumina Inc., San Diego, CA). Cluster formation of the library DNA templates was performed using the TruSeq PE Cluster Kit v3 (Illumina Inc., San Diego, CA) and the IlluminacBot workstation using conditions recommended by the manufacturer. Paired-end fifty base sequencing by synthesis was performed using TruSeq SBS kit v3 (Illumina Inc., San Diego, CA) on an IlluminaHiSeq 1000 using the manufacturer’s protocol. Cluster density per lane was 820–940 k/mm^2,^ and post-filter reads ranged from 148 to 218 million per lane.

### 4.3 Sequence analysis

Adapters were removed by the Cutadapt tool (Martin 2011) from all the samples. To assemble complete genomic sequences, we assessed the quality of the paired-end reads using FastQC (https://www.bioinformatics.babraham.ac.uk/projects/fastqc/); low base quality ends including untranslated regions were trimmed using the FASTX tool kit (http://hannonlab.cshl.edu/fastx_toolkit/). The remaining paired-end reads were aligned to an Indian strain isolated during an outbreak in 2006 (accession no. FJ000068), using Burrows-Wheeler Alignment (BWA) and default parameters. The reference sequence was the first genome that was submitted to Genbank after CHIKV re-emergence in India. The complete sequences of all samples have been submitted to Genbank (Table 1). An additional 753 CHIKV complete genomic sequences were retrieved from the ViPR (Pickett et al. 2012) that includes seventy-two Indian sequences and 681 sequences from other areas of the world to reveal the pattern of evolution in CHIKV both in India and in the global scenario. The flowchart of all the analyses performed in this study is represented in Supplementary Fig. S1.

### 4.4 Phylogenetic analysis

A dataset of 794 whole genome sequences were inferred for evolutionary relationship. All sequences were aligned using CLUSTALW software. The midpoint rooted phylogenetic tree analysis was done using the MEGAX (Kumar et al. 2018) software with ML methods, and bootstrap values were determined using 1000 replicates. Furthermore, a set of ninety-nine whole genomes of CHIKV (Indian isolates) reported from different regions of India between 2005 and 2016 was analysed for different evolutionary models using the MEGAX software to find the best-fit model of nucleotide substitutions for the sequence alignment before proceeding with the evolutionary analysis. The iTOL https://itol.embl.de/ (Interactive Tree Of Life (embl.de)) software has been used to visualize the phylogenetic tree. The neutrality of mutations in the sequences was determined using Tajima’s *D* test.

### 4.5 Nucleotide substitution rate, divergence times, recombination, and geographical structure of CHIKV

Bayesian Evolutionary Analysis Sampling Tree (BEAST2; Bouckaert et al. 2014) was used to calculate the phylogenetic relationships and the time to the most recent common ancestor for the coding region of all ninety-nine Indian CHIKV sequences. The necessary parameters and data were prepared in XML format using BEAUti version 2. The general time reversible (GTR) substitution model was selected with all estimated base frequencies and the gamma + invariant site heterogeneity model. BEAST2 was run using relaxed uncorrelated models. Multiple combinations of molecular clocks and coalescent models based on Bayes factor (>1) were run for the chain length of 100 million with a tree sampling every 10,000 chains, which was adequate to achieve stationarity. The time to the most recent common ancestor and the nucleotide substitutions per site per year were estimated. Using Tracer version 1.7.1, the Markov chain Monte Carlo steps and convergence of the runs were visualized as used to ensure stationarity was achieved, and TreeAnnotator version 2.6.0 was used to summarize the posterior tree distribution and generate the MCC tree. The phylogenetic tree with estimated divergence, posterior probability, 95 per cent highest posterior density, and variable timeline was generated and displayed using Figtree 1.4.2 (http://tree.bio.ed.ac.uk/software/figtree/). Bayesian hierarchical clustering of the phylogenetic tree was visualized and edited in DensiTree software of BEAST2 package. Determination of potential recombinant events, parental sequences, and localization of recombinant break points were performed triplet by triplet using the recombination detection program (RDP4; Martin et al. 2015) GENECONV, MaxChi, CHIMAERA, SiScan, and 3SEQ. The sequences were set to linear, and *P* values <0.05 were considered statistically significant.

Shannon entropy calculations were performed to identify the highly variable sites within each amino acid sequence with high entropy values. We used forty-one whole-genome sequences of CHIKV strains from our study for this analysis. The BioEdit 7.2 (https://bioedit.software.informer.com/7.2) stand-alone version was used to calculate the Shannon entropy of each amino acid site. A cut-off value of 0.2 was set, and the sites with values >0.2 were considered to be variable.

### 4.6 Variant analysis

Single-nucleotide polymorphism variants were identified using the Snippy variant identification tool (https://github.com/tseemann/snippy). Variants were visualized using the network and hierarchical clustering to facilitate the identification and analysis of similarities and differences arising from genome comparisons. Hierarchical clustering was done using the Morpheus software (https://software.broadinstitute.org/morpheus). Furthermore, a worldwide analysis was done using the MEGAX software.

### 4.7 Selection pressure analysis

The selection pressure acting on CHIKV genomes was investigated using the online facility at the web server http://www.datamonkey.org (Weaver et al. 2018). The complete data set of Indian strains (*n* = 99) with the ML statistical method has been used for this analysis. The ω ratios (non-synonymous (dN)/synonymous (dS)) substitutions) were calculated using methods such as SLAC, FEL, FUBAR, and MEME. Sites showing evidence of positive selection with a high statistical significance (*P* value <0.05 or Bayes factor >50) were considered to be under positive selection (Agarwal et al. 2016).

## Supplementary Material

veab074_Supp

## Data Availability

The data were obtained from the publically available ViPR. Samples from this study have been submitted to the National Center for Biotechnology Information.

## References

[R1] Agarwal A. et al. (2016) ‘Two Novel Epistatic Mutations (E1: K211E and E2: V264A) in Structural Proteins of Chikungunya Virus Enhance Fitness in *Aedes* *a**egypti*’, *Virology*, 497: S59–68. 10.1016/j.virol.2016.06.025 27423270

[R2] Arankalle V. A. et al. (2007) ‘Genetic Divergence of Chikungunya Viruses in India (1963–2006) with Special Reference to the 2005–2006 Explosive Epidemic’, *The Journal of General Virology*, 88: 1967–76. 17554030 10.1099/vir.0.82714-0

[R3] Barr K. L., and Vaidhyanathan V. (2019) ‘Chikungunya in Infants and Children: Is Pathogenesis Increasing?’ *Viruses*, 11: 294. 10.3390/v11030294 PMC6466311 30909568

[R4] Bouckaert R. et al. (2014) ‘BEAST 2: A Software Platform for Bayesian Evolutionary Analysis’, *PLoS Computational Biology*, 10: e1003537. 10.1371/journal.pcbi.1003537 PMC3985171 24722319

[R5] Cardoso F. D. et al. (2019) ‘Circulation of Chikungunya Virus East-Central-South Africa Genotype during an Outbreak in 2016–17 in Piaui State, Northeast Brazil’, *Revista do Instituto de Medicina Tropical de Sao Paulo*, 61: e57. 10.1590/S1678-9946201961057 PMC6792355 31618377

[R0005a] Chahar H. S. et al. (2009) ‘Co-infections with chikungunya virus and dengue virus in Delhi, India’, *Emerging infectious diseases*, 15: 1077–80. 19624923 10.3201/eid1507.080638 PMC2744227

[R7] Chen R. et al. (2016) ‘Comprehensive Genome Scale Phylogenetic Study Provides New Insights on the Global Expansion of Chikungunya Virus’, *Journal of Virology*, 90: 10600–11. 27654297 10.1128/JVI.01166-16 PMC5110187

[R8] Chhabra M. et al. (2008) ‘Chikungunya Fever: A Re-emerging Viral Infection’, *Indian**Journal of Medical Microbiology*, 26: 5–12. 18227590 10.4103/0255-0857.38850

[R9] Dash P. K. et al. (2007) ‘East Central South African Genotype as the Causative Agent in Reemergence of Chikungunya Outbreak in India’, *Vector-Borne and Zoonotic Diseases*, 7: 519–28. 18171110 10.1089/vbz.2007.7272

[R10] Dikid T. et al. (2013) ‘Emerging & Re-emerging Infections in India: An Overview’, *The Indian Journal of Medical Research*, 138: 19–31. 24056553 PMC3767269

[R11] Dwibedi B. et al. (2011) ‘Rapid Spread of Chikungunya Virus Infection in Orissa: India’, *The Indian Journal of Medical Research*, 133: 316–21. 21441687 PMC3103158

[R12] Forrester N. L. et al. (2012) ‘Genome-Scale Phylogeny of the Alphavirus Genus Suggests a Marine Origin’, *Journal of Virology*, 86: 2729–38. 22190718 10.1128/JVI.05591-11 PMC3302268

[R13] Fros J. J. et al. (2010) ‘Chikungunya Virus Nonstructural Protein 2 Inhibits Type I/II Interferon-Stimulated JAK-STAT Signaling’, *Journal of Virology*, 84: 10877–87. 20686047 10.1128/JVI.00949-10 PMC2950581

[R14] Ganesan V. K., Duan B., and Reid S. P. (2017) ‘Chikungunya Virus: Pathophysiology, Mechanism, and Modeling’, *Viruses*, 9: 368. 10.3390/v9120368 PMC5744143 29194359

[R15] Hyde J. L. et al. (2015) ‘The 5’ and 3’ Ends of Alphavirus RNAs—Non-coding Is Not Non-functional’, *Virus**Research*, 206: 99–107. 10.1016/j.virusres.2015.01.016 PMC4654126 25630058

[R16] Jain J. et al. (2017) ‘Clinical, Serological, and Virological Analysis of 572 Chikungunya Patients from 2010 to 2013 in India’, *Clinical Infectious Diseases*, 65: 133–40. 28379375 10.1093/cid/cix283

[R17] Jain J. et al. (2020) ‘Chikungunya Outbreaks in India: A Prospective Study Comparing Neutralization and Sequelae during Two Outbreaks in 2010 and 2016’, *The American Journal of Tropical Medicine and Hygiene*, 102: 857–68. 32067624 10.4269/ajtmh.19-0481 PMC7124918

[R18] Jenkins G. M. et al. (2002) ‘Rates of Molecular Evolution in RNA Viruses: A Quantitative Phylogenetic Analysis’, *Journal of Molecular Evolution*, 54: 156–65. 11821909 10.1007/s00239-001-0064-3

[R19] Jones J. E. et al. (2017) ‘Disruption of the Opal Stop Codon Attenuates Chikungunya Virus-Induced Arthritis and Pathology’, *mBio*, 8: e01456–17. 29138302 10.1128/mBio.01456-17 PMC5686535

[R20] Kaur N. et al. (2017) ‘Chikungunya Outbreak in Delhi, India, 2016: Report on Coinfection Status and Comorbid Conditions in Patients’, *New Microbes and**New**Infections*, 20: 39–42. 10.1016/j.nmni.2017.07.007 PMC5682881 29158907

[R21] Kumar S. et al. (2018) ‘MEGA X: Molecular Evolutionary Genetics Analysis Across Computing Platforms’, *Molecular Biology and Evolution*, 35: 1547–9. 29722887 10.1093/molbev/msy096 PMC5967553

[R22] Lanciotti R. S., and Lambert A. J. (2016) ‘Phylogenetic Analysis of Chikungunya Virus Strains Circulating in the Western Hemisphere’, *The American Journal of Tropical Medicine and Hygiene*, 94: 800–3. 26856917 10.4269/ajtmh.15-0375 PMC4824221

[R23] Langsjoen R. M. et al. (2018) ‘Chikungunya Virus Strains Show Lineage-Specific Variations in Virulence and Cross-Protective Ability in Murine and Nonhuman Primate Models’, *mBio*, 9: e02449–17. 29511072 10.1128/mBio.02449-17 PMC5844994

[R24] Leigh J. W., Bryant D., and Nakagawa S. (2015) ‘Popart: Full-Feature Software for Haplotype Network Construction’, *Methods in Ecology and Evolution*, 6: 1110–6.

[R25] Martin D. P. et al. (2015) ‘RDP4: Detection and Analysis of Recombination Patterns in Virus Genomes’, *Virus**Evolution*, 1: vev003. 10.1093/ve/vev003 PMC5014473 27774277

[R26] Martin M. (2011) ‘Cutadapt Removes Adapter Sequences from High-throughput Sequencing Reads’, *EMBnet. Journal*, 17.

[R28] Mounce B. C. et al. (2017) ‘Chikungunya Virus Overcomes Polyamine Depletion by Mutation of nsP1 and the Opal Stop Codon to Confer Enhanced Replication and Fitness’, *Journal of Virology*, 91: e00344–17. 28539441 10.1128/JVI.00344-17 PMC5512238

[R29] Munoz-Medina J. E. et al. (2018) ‘Evolutionary Analysis of the Chikungunya Virus Epidemic in Mexico Reveals Intra-host Mutational Hotspots in the E1 Protein’, *PLoS One*, 13: e0209292. 10.1371/journal.pone.0209292 PMC6294367 30550577

[R30] Nasci R. S. (2014) ‘Movement of Chikungunya Virus into the Western Hemisphere’, *Emerging Infectious Diseases*, 20: 1394–5. 25061832 10.3201/eid2008.140333 PMC4111178

[R31] Pavri K. (1986) ‘Disappearance of Chikungunya Virus from India and South East Asia’, *Transactions of the Royal Society of Tropical Medicine and Hygiene*, 80: 491. 10.1016/0035-9203(86)90358-5 3026069

[R32] Pickett B. E. et al. (2012) ‘ViPR: An Open Bioinformatics Database and Analysis Resource for Virology Research’, *Nucleic Acids**Research*, 40: D593–8. 10.1093/nar/gkr859 PMC3245011 22006842

[R33] Rezza G. (2014) ‘Dengue and Chikungunya: Long-distance Spread and Outbreaks in Naïve Areas’, *Pathogens and Global Health*, 108: 349–55. 25491436 10.1179/2047773214Y.0000000163 PMC4394667

[R34] Sahadeo N. S. D. et al. (2017) ‘Understanding the Evolution and Spread of Chikungunya Virus in the Americas Using Complete Genome Sequences’, *Virus**Evolution*, 3: 1. 10.1093/ve/vex010 PMC5413804 28480053

[R35] Sam I. C. et al. (2015) ‘Updates on Chikungunya Epidemiology, Clinical Disease, and Diagnostics’, *Vector-Borne and**Zoonotic**Diseases*, 15: 223–30. 10.1089/vbz.2014.1680 25897809

[R36] Sasmono R. T. et al. (2017) ‘Chikungunya Detection during Dengue Outbreak in Sumatra, Indonesia: Clinical Manifestations and Virological Profile’, *The American Journal of Tropical Medicine and Hygiene*, 97: 1393–8. 29016291 10.4269/ajtmh.16-0935 PMC5817737

[R37] Shah K. V., Gibbs C. J. Jr., and Banerjee G. (1964) ‘Virological Investigation of the Epidemic of Haemorrhagic Fever in Calcutta: Isolation of Three Strains of Chikungunya Virus’, *The Indian Journal of Medical Research*, 52: 676–83. 14195507

[R38] Silva L. A., and Dermody T. S. (2017) ‘Chikungunya Virus: Epidemiology, Replication, Disease Mechanisms, and Prospective Intervention Strategies’, *Journal of Clinical Investigation*, 127: 737–49. 28248203 10.1172/JCI84417 PMC5330729

[R39] Stapleford K. A. et al. (2016) ‘Whole-Genome Sequencing Analysis from the Chikungunya Virus Caribbean Outbreak Reveals Novel Evolutionary Genomic Elements’, *PLoS**Neglected Tropical Diseases*, 10: e0004402. 10.1371/journal.pntd.0004402 PMC4726740 26807575

[R40] Tsetsarkin K. A., and Weaver S. C. (2011) ‘Sequential Adaptive Mutations Enhance Efficient Vector Switching by Chikungunya Virus and Its Epidemic Emergence’, *PLoS**Pathogens*, 7: e1002412. 10.1371/journal.ppat.1002412 PMC3234230 22174678

[R41] Tsetsarkin K. A. et al. (2007) ‘A Single Mutation in Chikungunya Virus Affects Vector Specificity and Epidemic Potential’, *PLoS**Pathogens*, 3: e201. 10.1371/journal.ppat.0030201 PMC2134949 18069894

[R42] Villero-Wolf Y. et al. (2019) ‘Genomic Epidemiology of Chikungunya Virus in Colombia Reveals Genetic Variability of Strains and Multiple Geographic Introductions in Outbreak, 2014’, *Scientific Reports*, 9: 1–11. 31292455 10.1038/s41598-019-45981-8 PMC6620336

[R43] Volk S. M. et al. (2010) ‘Genome-scale Phylogenetic Analyses of Chikungunya Virus Reveal Independent Emergences of Recent Epidemics and Various Evolutionary Rates’, *Journal of Virology*, 84: 6497–504. 20410280 10.1128/JVI.01603-09 PMC2903258

[R44] Weaver S. et al. (2018) ‘Datamonkey 2.0: A Modern Web Application for Characterizing Selective and Other Evolutionary Processes’, *Molecular Biology and Evolution*, 35: 773–7. 29301006 10.1093/molbev/msx335 PMC5850112

[R45] Wimalasiri-Yapa B. et al. (2019) ‘Chikungunya Virus in Asia - Pacific: A Systematic Review’, *Emerging**Microbes**& Infections*, 8: 70–9. 10.1080/22221751.2018.1559708 PMC6455125 30866761

[R46] Wong K. Z., and Chu J. J. H. (2018) ‘The Interplay of Viral and Host Factors in Chikungunya Virus Infection: Targets for Antiviral Strategies’, *Viruses*, 10: 294. 10.3390/v10060294 PMC6024654 29849008

[R47] Xavier J. et al. (2019) ‘Circulation of Chikungunya Virus East/Central/South African lineage in Rio de Janeiro, Brazil’, *PLoS One*, 14: e0217871. 10.1371/journal.pone.0217871 PMC6559644 31185030

[R48] Yadav P. et al. (2003) ‘Genotyping of Chikungunya Virus Isolates from India during 1963–2000 by Reverse Transcription-Polymerase Chain Reaction’, *Acta Virologica*, 47: 125–7. 14524480

[R49] Yergolkar P. N. et al. (2006) ‘Chikungunya Outbreaks Caused by African Genotype, India’, *Emerging Infectious Diseases*, 12: 1580–3. 17176577 10.3201/eid1210.060529 PMC3290956

[R50] Yang Z. et al. (2000) ‘Codon-substitution Models for Heterogeneous Selection Pressure at Amino Acid Sites’, *Genetics*, 155: 431–49. 10790415 10.1093/genetics/155.1.431 PMC1461088

[R51] Zanotto P. M. et al. (1996) ‘A Reevaluation of the Higher Taxonomy of Viruses Based on RNA Polymerases’, *Journal of Virology*, 70: 6083–96. 8709232 10.1128/jvi.70.9.6083-6096.1996 PMC190630

[R52] Zeller H., Van Bortel W., and Sudre B. (2016) ‘Chikungunya: Its History in Africa and Asia and Its Spread to New Regions in 2013–2014’, *Journal of Infectious Diseases*, 214: S436–40. 27920169 10.1093/infdis/jiw391

