# Supplementary material for: Chikungunya virus molecular evolution in India since its re-emergence in 2005
Source: Virus Evol. 2021 Aug 25;7(2):veab074. doi: 10.1093/ve/veab074 (PMC8570154; doi:10.1093/ve/veab074)
Supplement: veab074_Supp [file veab074_supp.zip › Supplementary_material.docx]

**Supplementary Material**

Supplementary Table 1: Summary of BEAST analysis

| Summary Statistic | posteriorL9H8B1B1:N11 | likelihood | prior | treeLikelihood.MAFFT | TreeHeight | clockRate | mutationRate | gammaShape | kappa | CalibratedYuleModel | birthRateY | logP(mrca(sequenced)) | mrca.date(sequenced) |
| --- | --- | --- | --- | --- | --- | --- | --- | --- | --- | --- | --- | --- | --- |
| Mean | -34015.6792 | -34444.408 | 428.7288 | -34444.408 | 0.0281 | 1.6773 | 1 | 0.0256 | 4.8238 | 518.5128 | 798.2514 | -72.8948 | -0.0278 |
| Standard error of mean | 5.2343 | 0.2434 | 5.2431 | 0.2434 | 0.0013034 | 0.0927 | 0 | 0.00064017 | 0.005797 | 5.3043 | 43.7776 | 0.0312 | 0.0012896 |
| Standard deviation | 81.6419 | 8.1408 | 81.3861 | 8.1408 | 0.027 | 1.5298 | 0 | 0.0154 | 0.2063 | 82.2919 | 719.7221 | 0.6468 | 0.0267 |
| Variance | 6665.3947 | 66.2726 | 6623.7048 | 66.2726 | 0.00072764 | 2.3404 | 0 | 0.0002384 | 0.0426 | 6771.9549 | 518000 | 0.4183 | 0.00071138 |
| Median | -34014.0229 | -34444.0949 | 430.261 | -34444.0949 | 0.0194 | 1.2009 | 0 | 0.0245 | 4.8164 | 519.6477 | 572.943 | -72.688 | -0.0192 |
| Value range | [-34296.101, -33759.7223] | [-34481.6225, -34414.9483] | [141.3705, 692.2805] | [-34481.6225, -34414.9483] | [1.0988E-3, 0.3501] | [0.0635, 20.6571] | [1, 1] | [1.0013E-3, 0.0685] | [4.1766, 5.5486] | [235.1954, 790.9582] | [26.2814, 9056.2654] | [-80.8319, -72.2518] | [-0.3485, -0.0011] |
| Geometric mean | n/a | n/a | 420.5692 | n/a | 0.0196 | 1.1854 | n/a | 0.0195 | 4.8194 | 511.7476 | 565.2729 | n/a | n/a |
| 95% HPD interval | [-34171.3355, -33856.4599] | [-34460.3702, -34428.7507] | [269.5784, 584.6353] | [-34460.3702, -34428.7507] | [1.795E-3, 0.0819] | [0.0862, 4.7508] | n/a | [1.0013E-3, 0.052] | [4.4202, 5.1903] | [357.2634, 675.7051] | [46.8174, 2260.7299] | [-74.1838, -72.2675] | [-0.081, -0.0017] |
| Auto-correlation time (ACT) | 36995.6357 | 8044.7342 | 37353.8532 | 8044.7342 | 21014.0873 | 33016.8331 | n/a | 15471.7757 | 7106.4869 | 37394.2322 | 33299.3568 | 20965.4687 | 21040.1959 |
| Effective sample size (ESS) | 243.3 | 1118.8 | 240.9 | 1118.8 | 428.3 | 272.6 | n/a | 581.7 | 1266.5 | 240.7 | 270.3 | 429.3 | 427.8 |

Supplementary Table 2: Identified AA positions with high entropy value

| Amino Acid and Positions | Entropy Value |
| --- | --- |
| Position 24, H | 1.01 |
| Position 34, T | 1.03 |
| Position 56, P | 1.05 |
| Position 84, I | 1.05 |
| Position 92, G | 1.05 |
| Position 110,R | 1.01 |
| Position 207, Y | 1.02 |
| Position 1694, S | 1.14 |
| Position 1706, V | 1.02 |
| Position 3887, - | 1 |
| Position 3958,- | 1.06 |
| Position 3959, - | 1.14 |
| Position 3974, - | 1.1 |

Supplementary Table 3: Non-synonymous mutations that distinguish CHIKV sequences analyzed in the study.

| Gene | Mutation Positions | Substitution | Reference |
| --- | --- | --- | --- |
| nsP1 | 103 | V A | From this study |
|  | 230 | G R | Mounce et al., 2017 |
|  | 314 | M L | Kumar et al., 2014 |
|  | 491 | R W | From this study |
| nsP2 | 130 | H Y | Somlor et al., 2017 |
|  | 145 | E D | Lindh et al., 2019 |
|  | 374 | Y H | From this study |
|  | 442 | N D | From this study |
|  | 446 | T S | From this study |
|  | 689 | P S | From this study |
|  | 764 | F L | From this study |
| nsP3 | 258 | N K | From this study |
|  | 459 | T M | From this study |
|  | 501 | S L | From this study |
| nsP4 | 55 | S N | Lindh et al., 2019 |
|  | 85 | R G | Lindh et al., 2019 |
|  | 254 | A T | From this study |
|  | 514 | T I | From this study |
|  | 563 | I V | From this study |
| C | 23 | S P | From this study |
|  | 27 | I V | From this study |
|  | 58 | Q R | From this study |
| E3 | 42 | V I | Zhang et al., 2018 |
|  | 59 | P S | From this study |
| E2 | 55 | G R | Jain et al., 2020 |
|  | 73 | H Y | Jain et al., 2020 |
|  | 221 | K R | Galan-Huerta et al., 2019 |
|  | 264 | V A | Agarwal et al., 2016 |
|  | 267 | R M | From this study |
|  | 312 | M T | From this study |
|  | 351 | H R | Rodas et al., 2016 |
|  | 386 | A V | From this study |
| E1 | 211 | K E | Ankita et al., 2016 |
|  | 269 | V M | Tsetsarkin et al., 2014 |
|  | 288 | T I | Pouriayevali et al., 2019 |
|  | 317 | I V | Harsha et al., 2020 |
| 6K | 54 | V I | From this study |

Figure Legends

Supplementary Figure 1**:** Pictorial representation of the workflow followed for whole genome sequencing analysis.

Supplementary Figure 2: An unrooted Maximum likelihood phylogenetic tree of chikungunya virus strains using complete genome sequences representing global diversity. A total of 794 sequences have been taken for this study. Nodes in red colour represent strains from present study while purple colour nodes represent data from VIPR database. Green colour on branches is showing bootstrap value (>70%), Highlighted clades are showing Indian sequences from 2016 clustering together with other countries.

Supplementary figure 3 : MCC phylogeny based on Indian CHIKV strains(n=99) including 41 sequences derived from the present study. Taxon labels include isolation country name/year of sample collection/state/sample number. Green colour of branches represent isolates from Mumbai, blue from Delhi and black from other states of India. Red colour of nodes represent isolates from Delhi and violet from Mumbai of present study while black colour of nodes represent isolates from database. Numbers on branches showing node ages.
