# Supplementary figures and images for: Chikungunya virus molecular evolution in India since its re-emergence in 2005
Source: Virus Evol. 2021 Aug 25;7(2):veab074. doi: 10.1093/ve/veab074 (PMC8570154; doi:10.1093/ve/veab074)

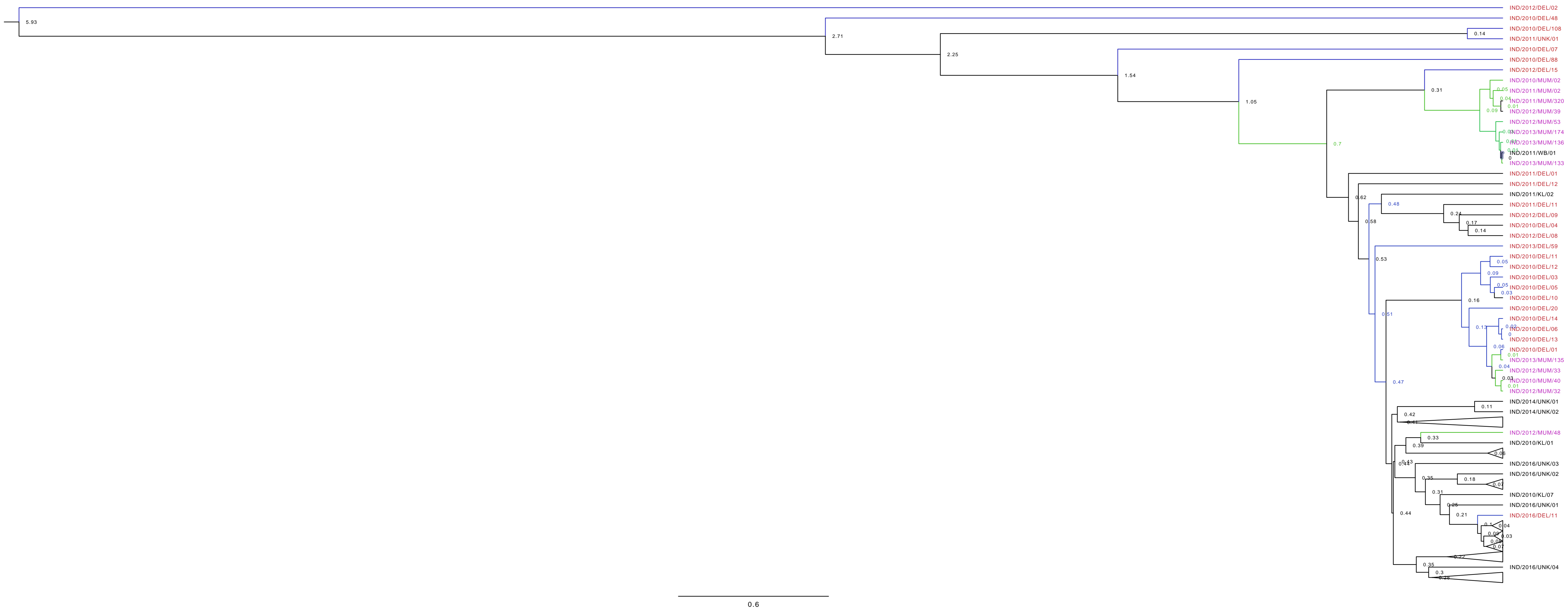

Supplement: veab074_Supp [file veab074_supp.zip › Supplementary_figure_3.pdf]

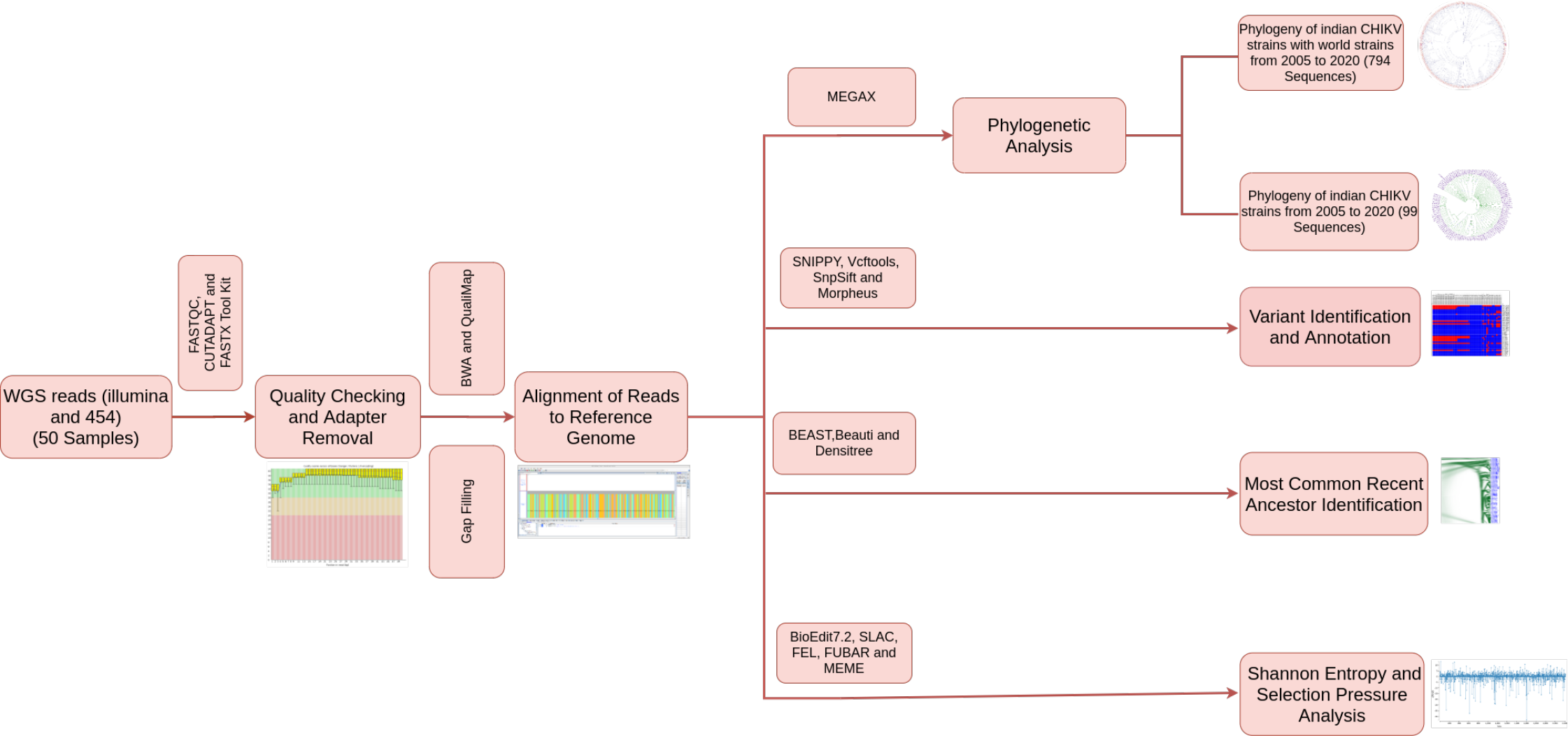

Supplement: veab074_Supp [file veab074_supp.zip › Supplementary_figure1.pdf]

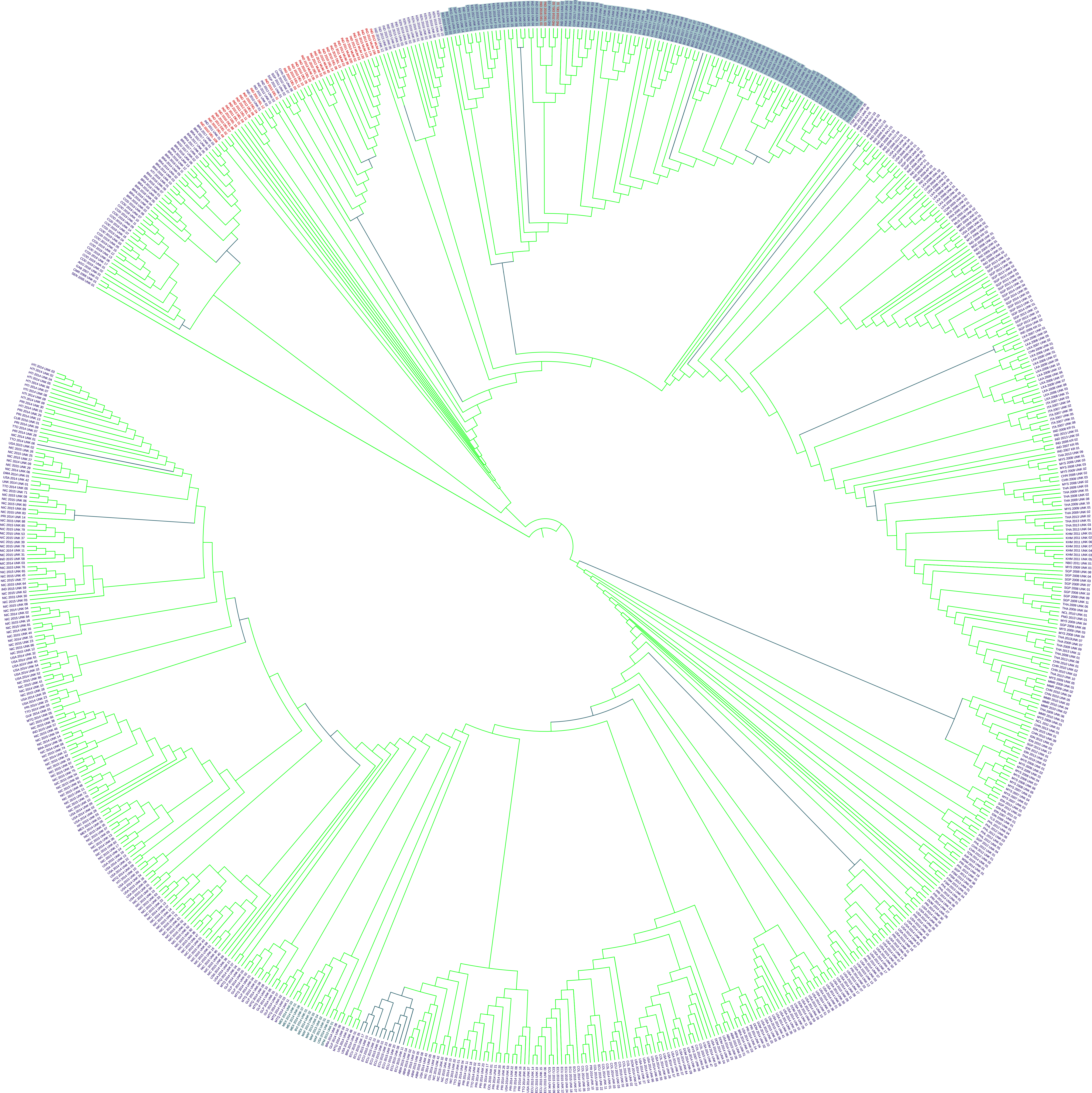

Supplement: veab074_Supp [file veab074_supp.zip › Supplementary_figure2.pdf]
